# High-Level Conversion of l-lysine into Cadaverine by *Escherichia coli* Whole Cell Biocatalyst Expressing *Hafnia alvei* l-lysine Decarboxylase

**DOI:** 10.3390/polym11071184

**Published:** 2019-07-14

**Authors:** Hee Taek Kim, Kei-Anne Baritugo, Young Hoon Oh, Kyoung-Hee Kang, Ye Jean Jung, Seyoung Jang, Bong Keun Song, Il-Kwon Kim, Myung Ock Lee, Yong Taek Hwang, Kyungmoon Park, Si Jae Park, Jeong Chan Joo

**Affiliations:** 1 Bio-Based Chemistry Research Center, Advanced Convergent Chemistry Division, Korea Research Institute of Chemical Technology, P.O. Box 107, 141 Gajeong-ro, Yuseong-gu, Daejeon 34114, Korea; 2 Division of Chemical Engineering and Materials Science, Ewha Womans University, 52 Ewhayeodae-gil, Seodaemun-gu, Seoul 03760, Korea; 3 Department of Biological and Chemical Engineering, Hongik University, 2639 Sejong-ro, Sinan-ri, Jochiwon-eup, Sejong-si 30016, Korea; 4 Bioprocess R&D Center, DAESANG Corp., Icheon-si, Gyeonggi-do 17384, Korea; 5 Lotte Chemical, 115 Gajeongbuk-ro, Yuseong-gu, Daejeon 34110, Korea; 6 Department of Chemistry, KAIST, 291, Daehak-ro, Yuseong-gu, Daejeon 34141, Korea

**Keywords:** cadaverine, IPTG- and PLP-free whole cell biocatalyst reaction, lysine decarboxylase, *Hafnia alvei*, polyamide 510

## Abstract

Cadaverine is a C5 diamine monomer used for the production of bio-based polyamide 510. Cadaverine is produced by the decarboxylation of l-lysine using a lysine decarboxylase (LDC). In this study, we developed recombinant *Escherichia coli* strains for the expression of LDC from *Hafnia alvei*. The resulting recombinant XBHaLDC strain was used as a whole cell biocatalyst for the high-level bioconversion of l-lysine into cadaverine without the supplementation of isopropyl β-d-1-thiogalactopyranoside (IPTG) for the induction of protein expression and pyridoxal phosphate (PLP), a key cofactor for an LDC reaction. The comparison of results from enzyme characterization of *E. coli* and *H. alvei* LDC revealed that *H. alvei* LDC exhibited greater bioconversion ability than *E. coli* LDC due to higher levels of protein expression in all cellular fractions and a higher specific activity at 37 °C (1825 U/mg protein > 1003 U/mg protein). The recombinant XBHaLDC and XBEcLDC strains were constructed for the high-level production of cadaverine. Recombinant XBHaLDC produced a 1.3-fold higher titer of cadaverine (6.1 g/L) than the XBEcLDC strain (4.8 g/L) from 10 g/L of l-lysine. Furthermore, XBHaLDC, concentrated to an optical density (OD_600_) of 50, efficiently produced 136 g/L of cadaverine from 200 g/L of l-lysine (97% molar yield) via an IPTG- and PLP-free whole cell bioconversion reaction. Cadaverine synthesized via a whole cell biocatalyst reaction using XBHaLDC was purified to polymer grade, and purified cadaverine was successfully used for the synthesis of polyamide 510. In conclusion, an IPTG- and PLP-free whole cell bioconversion process of l-lysine into cadaverine, using recombinant XBHaLDC, was successfully utilized for the production of bio-based polyamide 510, which has physical and thermal properties similar to polyamide 510 synthesized from chemical-grade cadaverine.

## 1. Introduction

The bio-based production of industrial platform chemicals and fuels from renewable resources in biorefineries is a promising, sustainable alternative to current petroleum-based chemical production processes [1,2,3,4,5,6,7]. Biorefineries have been successfully developed for the conversion of a broad range of biomass feedstocks, such as lignocellulosic hydrolysates, algal residue, and recalcitrant coal, into chemicals with properties comparatively similar to petrochemical-based products [8,9,10,11,12,13,14,15,16,17,18,19,20,21,22]. In biorefineries, the microbial fermentation of biomass-derived sugars has successfully produced numerous value-added biochemicals such as amino acids, dicarboxylic acids, amino carboxylic acids, and short chain diamines [23,24,25,26,27,28,29,30,31,32,33,34,35,36,37,38,39,40,41,42,43]. These biochemicals are considered platform chemicals which may be used for the production of bio-based polymers, such as polylactic acid (PLA), polybutylene succinate (PBS), and polyamides 54, 56, and 510 [44,45]. Polyamides have been successfully synthesized from fermentation-derived bio-based monomers including diamines, dicarboxylic acids, and amino carboxylic acids. The material properties of these bio-based polyamides are comparable to those of petrochemical-based polyamides and thus considered as green plastics in various industrial applications. The broad range of carbon numbers and functional groups in the bio-based monomers has enabled the sustainable production of polyamides with versatile material properties [1,2,3,4,5,22,23,24,25,26,27,28,29,30,31,32,33,34,35,36,37,38,39,40,41,42,43]. Currently, various bio-based polyamides, such as polyamide 4, polyamide 510, and polyamide 65, have been developed for application in areas such as apparels, food packaging, automobiles, electronics, and the textile industry [4,30,34,35,36,37,38,39,40].

Cadaverine is a C5 diamine that has a similar structure to hexamethylene-diamine, a monomer which is used in the petrochemical-based production of polymers like polyurethanes and non-isocyanate polyurethanes (NIPU) [15]. The bio-based production of cadaverine has been extensively studied because of its application as a co-monomer for production of various bio-nylons. Cadaverine can be produced by decarboxylation of l-lysine using an l-lysine decarboxylase (LDC) [3,4,23]. The direct fermentative production of cadaverine from renewable resources such as starch and lignocellulosic hydrolysates has been extensively studied using recombinant strains of *Escherichia coli* and *Corynebacterium glutamicum* [4,23,24,25,26,27,28,29,30,31,32,33,34,35,36]. The biosynthesis of cadaverine in *E. coli* occurs naturally due to two endogenous lysine decarboxylases; constitutive LdcC and inducible CadA [23]. During the early stages of cadaverine biosynthesis, 9.61 g/L of cadaverine was produced in fed-batch fermentation using recombinant *E. coli* XQ56 with plasmid-based overexpression of the *cadA* gene under a strong *tac* promoter and dihydrodipicolinate synthase (*dapA*) under a strong *trc* promoter. Key enzymes related to cadaverine utilization pathways, putrescine/cadaverine aminopropyltransferase, spermidine acetyltransferase, glutamate-putrescine/glutamate-cadaverine ligase, and putrescine/cadaverine aminotransferase (ΔspeE, ΔspeG, ΔpuuA, and ΔygjG), were deleted [36]. To improve the direct fermentative production of cadaverine, the recombinant *C. glutamicum* strains were developed via the heterologous expression of either the *E. coli cadA* or *ldcC* gene [4,23,24,25,26,27,28,29,30,31,32,33,34,35]. Most recombinant *C. glutamicum* strains produced higher titers of cadaverine from biomass-derived sugars than recombinant *E. coli* due to the natural capability of *C. glutamicum* for overproducing l-lysine, an important precursor of cadaverine, and the absence of related pathways for cadaverine utilization [4,23]. In fact, the highest titer of 103.8 g/L of cadaverine was produced from glucose by a fed-batch cultivation of recombinant *C. glutamicum* with the chromosomal integration of *E. coli ldcC* gene under a strong promoter, P_H30_, at the lysine exporter site (*lysE*) [35]. However, the direct fermentative production of cadaverine from glucose is limited by low titers (9.61–103.8 g/L) and the complicated nature of downstream processes involved in the purification of cadaverine [35,36]. Therefore, whole cell bioconversion is being explored as an alternative process for the economical production of cadaverine, because the substrate is directly converted into the target product using recombinant microorganisms as biocatalysts. This approach has already been used for the high-level production of polyamide monomers such as gamma-aminobutyric acid (GABA), 5-aminovaleric acid (5-AVA), and cadaverine [38,39,40,43]. It has been reported that 5.46 g/L of GABA, a polyamide 4 monomer, can be produced from 10 g/L of l-glutamate via whole cell bioconversion using recombinant *E. coli* strains expressing a glutamate decarboxylase [39]. The whole cell bioconversion of 120 g/L of l-lysine into 91 g/L of 5-aminovaleric acid was achieved by using recombinant *E. coli* expressing delta-aminovaleramidase and l-lysine 2-monooxygenase from *Pseudomonas putida* (*davBA*) [38]. The high-level production of 134 g/L of cadaverine was produced from 193 g/L of l-lysine via whole cell bioconversion using recombinant *E. coli* strains with an overexpression of the *ldcC* gene [40]. The whole cell bioconversion of l-lysine into cadaverine utilizing recombinant *E. coli* JM109 expressing *cadA* produced 69 g/L cadaverine in six hours with a productivity of 11.5 g/L/h [41]. Moreover, the whole cell bioconversion of l-lysine into cadaverine by employing an *E. coli* strain BL21 expressing *cadA* along with *cadB* encoding l-lysine/cadaverine antiporter produced cadaverine as high as 221 g/L with a molar yield of 92% when 8 g/dry cell weight per L of recombinant *E. coli* cells were employed as a catalyst for the fed-batch bioconversion of 344 ± 5 g/L of l-lysine [42].

**Figure 1:**
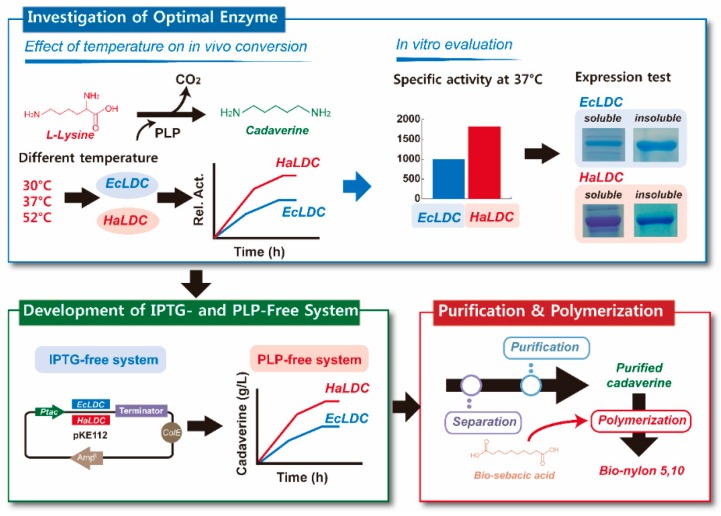
Schematic diagram for proposed production of bio-nylon 510 using purified cadaverine obtained from the pyridoxal pyrophosphate (PLP)- and isopropyl β-d-1-thiogalactopyranoside (IPTG)-free whole cell bioconversion of l-lysine, which is catalyzed by recombinant *E. coli* strains expressing a lysine decarboxylase from *H. alvei* and *E. coli*.

Previously, we reported the development of *E. coli* whole cell biocatalysts for cadaverine production from l-lysine by analyzing five different l-lysine decarboxylases from *E. coli*, *Ralstonia eutropha*, *Pseudomonas aeruginosa*, and *Pseudomonas putida* [40]. An l-lysine decarboxylase with a high activity can be a good candidate for the preparation of a whole cell biocatalyst. Among these LDC enzymes, an *E. coli* l-lysine decarboxylase encoded by *ldcC* was found to be the most promising LDC for the production of cadaverine from l-lysine under optimized reaction conditions, based on results related to substrate pH, substrate concentrations, buffering conditions, and biocatalyst concentrations [40,43]. Recently, a *Hafnia alvei* l-lysine decarboxylase was reported to have higher l-lysine decarboxylase activity than constitutive LdcC and inducible CadA from *E. coli* [46]. Therefore, the cadaverine production capability of the recombinant strain harboring an l-lysine decarboxylase genes from *E. coli* and *Hafnia alvei* can be investigated to develop an efficient process for cadaverine production. In the current study, we developed recombinant *E. coli* strains for the expression of a *Hafnia alvei* lysine decarboxylase (*ldcC*) in order to enhance the titer as well as the yield of cadaverine from the bioconversion of l-lysine in a reaction medium, without the additional use of inducers and cofactors, such as isopropyl β-d-1-thiogalactopyranoside (IPTG) and pyridoxal pyrophosphate (PLP), respectively. Furthermore, purified bio-based cadaverine, produced via the whole cell reaction described above, was then used for polymerization with sebacic acid to synthesize bio-based polyamide 510 (Figure 1). The whole cell bioconversion process described in this study provides a more economical process for commercial cadaverine production, because the system efficiently converts l-lysine into cadaverine without IPTG and PLP supplementation, which eliminates additional costs associated with such supplementation.

## 2. Materials and Methods 

### 2.1. Bacterial Strains, Plasmids, Genes, and Chemicals

**Figure 2:**
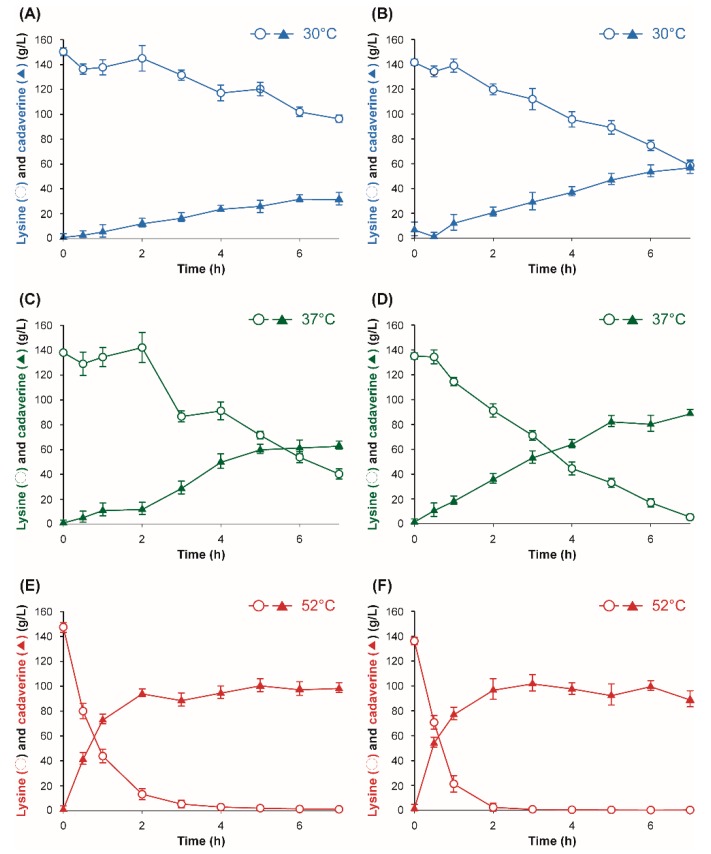
Time profiles of l-lysine consumption and cadaverine production by recombinant *E. coli* BL21 (DE3) expressing an *E. coli* lysine decarboxylase (LDC) (**A**,**C**,**E**) and *H. alvei* LDC (**B**,**D**,**F**), respectively. The whole cell biocatalysts were concentrated to OD_600_ = 10 and were resuspended in a sodium acetate buffer (pH 6.0) supplemented with 0.1 mM of PLP and 0.05 mM of IPTG for the conversion of 1 M of chemical grade l-lysine substrate (Symbols: open circle, l-lysine concentration; filled triangle, cadaverine concentration).

**Figure 3:**
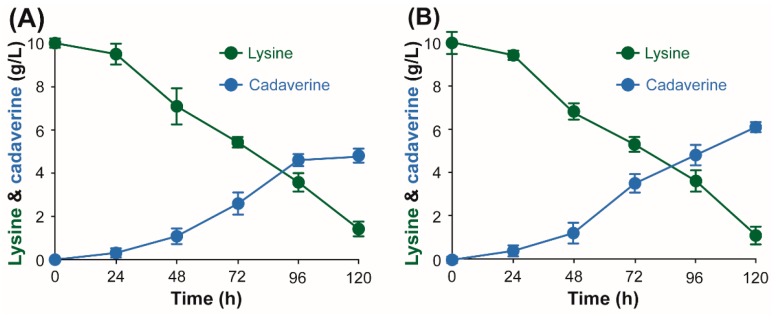
Time profiles of l-lysine consumption and cadaverine synthesis by recombinant *E. coli* XBEcLDC (**A**) and XBHaLDC (**B**) harboring *E. coli ldcC* gene and *H. alvei ldcC*, respectively. Recombinant strains were cultivated for 24 h in anLB medium and transferred into a 250 mL flask with a PLP-free MR medium with 20 g/L of glucose and 10 g/L of chemical-grade l-lysine for further cultivation at 37 °C (Symbols: filled circle, l-lysine concentration; open circle, cadaverine concentration).

**Figure 4:**
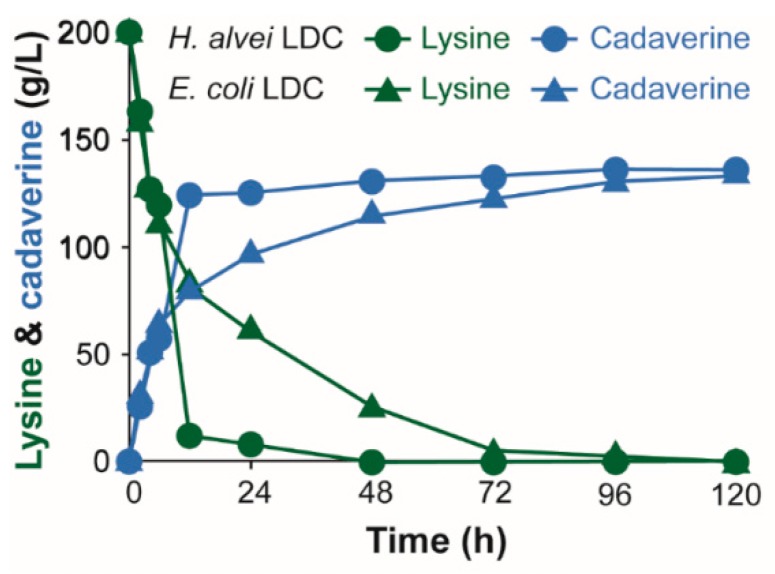
Time profiles of l-lysine consumption and cadaverine synthesis by recombinant *E. coli* XL1-Blue strain harboring *E. coli ldcC* gene and *H. alvei ldcC* genes in shake flask scale whole cell bioconversion reactions. A recombinant *E. coli* strains seed inoculum (OD_600_ = 50) was prepared as whole cell biocatalysts for the conversion of 200 g/L of industrial grade l-lysine to cadaverine. Data represent mean values from three independent experiments with corresponding standard deviations. (Symbols: filled circle, l-lysine concentration; open circle, cadaverine concentration).

The bacterial strains and plasmids used in this study are listed in Table 1. *E. coli* XL1-Blue (Stratagene Cloning Systems, La Jolla, CA, USA) was used for general gene cloning. For enzyme characterization experiments, *ldc* genes were expressed in *E. coli* BL21 (DE3) using pET vectors. A recombinant *E. coli* XL1-Blue strain expressing *ldc* genes from *H. alvei* and *E. coli* were used for whole cell bioconversion reactions. The plasmid pKE112-MCS with a strong *tac* promoter was used for the IPTG-free expression of *ldc* genes [38,40]. A 1.5 M substrate stock solution prepared from chemical grade l-lysine (St. Louis, MO, USA) was used for indicated whole cell bioconversion reactions (Figure 2 and Figure 3). An industrial grade of l-lysine was provided by DAESANG Corp. (Gunsan, Korea). A 2.73 M l-lysine stock solution (400 g/L of l-lysine) prepared from the industrial grade l-lysine was appropriately diluted to indicated concentrations in the reaction mixture (Figure 4).

### 2.2. Plasmid Construction

All DNA manipulations were performed following standard procedures. A polymerase chain reaction (PCR) was performed with a C1000 Thermal Cycler (Bio-Rad, USA). Primers used in this study were synthesized at Bioneer (Daejeon, Korea); (Table S1). *E. coli ldcC* (GenBank accession number NP_414728.1) and *H. alvei ldcC* (GenBank accession number X03774.1) were cloned into pKE112-MCS vector at *Bam*HI and *Sbf*I sites to construct pKE112-EcLdcC and pKE112-HaLdcC, respectively. For enzyme purification, *E. coli* and *H. alvei ldcC* genes were prepared using appropriate primers for cloning each gene into pET22b(+) at *Xba*I and *Xho*I sites, respectively (Table S1). For the whole cell bioconversion of l-lysine into cadaverine at 30 °C, 37 °C, and 52 °C, plasmids used to express *E. coli* and *H. alvei ldc* genes were constructed by the insertion of the respective *ldc* at *Hind*III and *Xho*I, of pET24ma(+) [43].

### 2.3. Purification of E. coli and H. alvei l-lysine Decarboxylase for In Vitro Analysis of Enzyme Activities

*H. alvei* and *E. coli* l-lysine decarboxylases were purified and subjected to enzymatic characterization. The purification of LDC enzymes was performed as previously described [47]. *E. coli* BL21 (DE3) was transformed with engineered pET vectors for the expression of LDC from *E. coli* and *H. alvei* (Table 1). The resulting recombinant strains were cultivated in 100 mL of a Luria Bertani (LB) medium in a 250 mL flask for 24 h at 37 °C and 250 rpm. The overexpression of *E. coli* and *H. alvei* LDC enzymes was induced by the addition of 0.05 M IPTG. After 24 h of induction at 20 °C, cells were centrifuged at 4000 rpm for 20 min at 4 °C and resuspended in a lysis buffer (50 μM of pyridoxal phosphate (PLP), 50 mM of NaH_2_PO_4_, 300 mM of NaCl, and 10 mM of imidazole: pH 8). Then, the resuspended cells were sonicated using an ultrasonicator (Vibra Cell, Soncis Scientific, Newtown, CT, USA) for 20 min on ice (5 s on, 8 s off). The cell lysates were centrifuged, and the supernatant was incubated with an Ni-NTA affinity resin for 30 min. The Ni-NTA resin was washed three times with a wash buffer (50 mM of NaH_2_PO_4_, 300 mM of NaCl, and 20 mM of imidazole: pH 8). Then, His-tagged LDCs were eluted with an elution buffer (50 mM of NaH_2_PO_4_, 300 mM of NaCl, and 250 mM of imidazole: pH 8). The in vitro enzyme activity of purified LDC enzymes was measured in 96-well plates using a 2,4,6-trinitrobenzenesulfonic acid (TNBS)-based assay as previously described [46]. Reaction mixtures contained 0.2 mM of PLP, l-lysine (0.1–20 mM), and the LDC enzyme (0.2 ug/L) in a final volume of 200 uL of a 0.05 mM sodium citrate buffer. One unit of l-lysine decarboxylase activity was defined as the amount of enzyme producing 1μmol of cadaverine per min at 37 °C.

### 2.4. Whole Cell Bioconversion of l-Lysine into Cadaverine by the Recombinant E. coli Strains Expressing E. coli and H. alvei l-Lysine Decarboxylase

Overnight cultures of recombinant BL21 (DE3) and *E. coli* XL1-Blue strains expressing *E. coli ldcC* or *H. alvei ldcC* were incubated at 37 °C, 250 rpm in an LB medium. In order to prepare whole cell biocatalysts for cadaverine production with PLP supplementation, 10 mL of overnight cultures of *E. coli* BL21 (DE3) harboring *E. coli ldcC* or *H. alvei ldcC* were transferred to 500 mL of an LB medium in 2 L baffled flasks. Cells were induced with 0.05 mM of IPTG after reaching OD_600_ between 0.5 and 0.6 and were incubated for 24 h at 20 °C and 250 rpm. Cells were collected and diluted with a sodium acetate buffer to obtain OD_600_ of 10 for whole cell bioconversions at 30, 37, or 52 °C with PLP supplementation (Figure 2). 1.5 M of chemical grade l-lysine were added to reaction mixtures to obtain a final concentration of 1 M of l-lysine substrate. The reactions were stopped by heating at 100 °C for 5 min. For cadaverine production under IPTG and PLP-free conditions (Figure 3 and Figure 4), the overnight cultures of *E. coli* XL1-Blue *E. coli ldcC* or *H. alvei ldcC* were first transferred to 100 mL of a PLP-free MR medium in a 250 mL flask with 20 g/L of glucose and 10 g/L (68 mM) of chemical grade l-lysine, and the cells were incubated at 37 °C and 250 rpm (Figure 3). For the whole cell bioconversion of industrial grade l-lysine (Figure 4), 10 mL of overnight cultures of *E. coli* XL1-Blue harboring *E. coli ldcC* or *H. alvei ldcC* were used to inoculate 500 mL of a PLP-free MR medium in 2 L baffled flasks which are incubated for 24 h at 37 °C, 250 rpm, and cells were collected and diluted to obtain OD_600_ of 50 (Figure 4). The PLP-free MR medium (pH 7.0) contained: 6.67 g/L of KH_2_PO_4_, 4 g/L of (NH_4_)_2_HPO_4_, 0.8 g/L of MgSO_4_·7H_2_O, 0.8 g/L of citric acid, and 5 mL/L of a trace metal solution. The trace metal solution (0.5 M of HCl) contained 10 g/L of FeSO_4_·7H_2_O, 2 g/L of CaCl_2_, 2.2 g/L of ZnSO_4_·7H_2_O, 0.5 g/L of MnSO_4_·4H_2_O, 1 g/L of CuSO_4_·5H_2_O, 0.1 g/L of (NH_4_)_6_Mo_7_O_24_·4H_2_O, and 0.02 g/L of Na_2_B_4_O_7_·10H_2_O. Glucose and MgSO_4_·7H_2_O were separately sterilized in an autoclave. 2.73 M of an industrial grade l-lysine stock solution were added to reaction mixtures to obtain a final concentration of a 1.37 M l-lysine substrate (200 g/L). Both 1.5 M of chemical grade l-lysine and 2.73 M of industrial grade l-lysine stock solutions were filter-sterilized using a 0.22 uM polyethersulfone (PES) membrane. An appropriate amount of 1 N of HCl was added to the substrate solutions to adjust the pH to 6.8 prior to filtration. Ampicillin (50 μg/mL) or kanamycin (15 μg/mL) was added to the medium for selection of bacteria with plasmids.

### 2.5. Purification of Crude Cadaverine Soluton from the Whole Cell Bioconversion of l-lysine

The purification of the crude cadaverine solution, obtained from the whole cell bioconversion of l-lysine, was performed according to a previously reported procedure [35]. An appropriate amount of NaOH was added to the crude cadaverine solution to adjust the pH to 14 in order to improve the efficiency of solvent extraction. Next, equal volumes of pH-adjusted crude cadaverine solution and chloroform were mixed in order to extract the cadaverine into the chloroform phase. Then, the mixed solution of cadaverine and chloroform was heated for 2 h at 55 °C. Following 2 h, the chloroform phase was collected in a round-bottom flask, and the water phase containing cadaverine was subjected to the same extraction procedure described above. The collected chloroform phase was concentrated using a rotary evaporator at 175 rpm and 60 °C. Finally, cadaverine of high purity (>99.5%) was obtained by fractional distillation using a laboratory distillation apparatus. Distillation was performed at 25−120 mbar and 120−135 °C [35].

### 2.6. Polymerization of Purified Cadaverine for the Synthesis of Bio-Based Polyamide 5,10

Polymerization for the synthesis of bio-based polyamide 510 was conducted by using equimolar purified cadaverine (65 g, 636 mmol) and sebacic acid (126 g, 623 mmol). For the polymerization reaction, a monomer solution was dissolved in deionized water (170 g), and a total of 361 g of the reaction solution was added to a 1 L autoclave reactor. Sodium hypophosphite monohydrate 13.2 mg (0.12 mmol, 79 ppm of final polymer) was used as reaction catalyst, and nitrogen gas was flushed to remove oxygen in the reactor. Polyamide 510 salt was obtained by increasing the temperature to 140 °C for 30 min. A pre-polymerization reaction was conducted at 215 °C and 17.2 bar, and the water vapor was slowly removed to maintain the constant pressure for 45 min. The pre-polymerization step was completed by the gradual reduction of pressure to atmospheric pressure. In the polymerization step, the reactor temperature was first heated to 270 °C. The reactor was stirred for 80 min at 1 atm and then further stirred for 10 min under a complete vacuum condition. Finally, polyamide 510 was obtained via extrusion under nitrogen pressure.

### 2.7. Analytical Procedures

Cadaverine and l-lysine concentrations were determined using HPLC fitted with an Optimapak C18 column (RStech, DaeJeon, Korea) as previously described [35,40,43]. For a comparison of the expression levels of the l-lysine decarboxylase enzymes from *H. alvei* and *E. coli*, a bradford assay and 10% SDS-PAGE analysis were conducted using recombinant XBHaLDC, XBEcLDC, BL21HaLDC, and BL21EcLDC strains (Figures S1 and S2). To characterize bio-based polyamide 510 produced from purified cadaverine, a proton nuclear magnetic resonance (1H NMR, 261DD2 (500 MHz), Agilent) analysis was conducted using bio-based polyamide 510 dissolved in hexa fluoro-2-propanol (HFIP) and CDCl_3_. Differential scanning calorimetry (DSC, Q200, TA Instruments, New Castle, DE, USA) was used to measure melting temperature (T_m_) and crystallization temperature (T_c_). DSC analysis was performed by melting polyamide 510 gradually at a rate of 10 °C/min. Next, the sample was cooled and reheated at the same rate from 30 to 250 °C. The degradation temperature (T_d_) of polyamide 510 was determined using thermogravimetric analysis (TGA, Q2950, TA Instruments). TGA analysis was conducted via the application of heat from 40 to 900 °C at 20 °C/min under N_2_ [35].

## 3. Results and Discussion

### 3.1. Characterization of Lysine Decarboxylase from E. coli and H. alvei

In order to develop an efficient and economic whole cell conversion system for cadaverine production, the recombinant biocatalyst should be active at moderate temperatures of 30 and 37 °C. We evaluated the activity of the lysine decarboxylase from *E. coli* and *H. alvei* at 30 and 37 °C, because these temperatures are generally optimal for cultivation of recombinant *E. coli* biocatalyst. We also evaluated bioconversion at 52 °C because it was previously reported as optimal for *E. coli* LDC activity (Figure 2) [40,43,48]. Though the reported optimal temperature for *H. alvei* LDC is 37 °C, a lysine decarboxylase from both *H. alve*i and *E. coli* was able to achieve a maximal rate of bioconversion at a higher temperature of 52 °C [47]. After 7 h of reaction, *H. alvei* LDC converted 146 g/L (1 M) of l-lysine into 89 g/L of cadaverine, while *E. coli* LDC produced 98 g/L. When the reactions were performed at 30 and 37 °C, *H. alvei* LDC produced 57 and 89 g/L of cadaverine, respectively. These values were 81% and 42% higher than the cadaverine titer produced by *E. coli* LDC, which were 32 and 62 g/L at 30 and 37 °C, respectively. These results were similar to previously reported optimal temperatures for *H. alvei* and *E. coli* constitutive lysine decarboxylases, which were 37 and 52 °C, respectively [43,46,47,48,49,50]. Based on these results, the system harboring a lysine decarboxylase from *H. alvei* was identified as a superior enzyme for industrial processing at 37 °C due to the conversion temperature being similar to catalyst cultivation temperatures, which, in turn, positively affects the overall operating cost (Figure 2).

An in vitro enzyme assay was conducted to the evaluate enhanced l-lysine conversion by a lysine decarboxylase from *H. alvei*. In addition, SDS-PAGE was performed to analyze protein expression (Figure S1). Experiments for characterization of LDC enzyme kinetics using purified *H. alvei* LDC and *E. coli* LDC were performed, but no saturation for l-lysine (0.1–20 mM) was observed (Figure S3). In the presence of 20 mM l-lysine at 37 °C, the specific activity of *H. alvei* LDC (1746 U/mg protein) was 1.7-fold higher than that of *E. coli* LDC (1021 U/mg protein). The specific activities of a purified lysine decarboxylase from *E. coli* and *H. alvei* at 37 °C were comparable to previously reported values, which were 1500 U/mg protein and 2466 U/mg protein, respectively [46]. Moreover, SDS-PAGE analyses showed that the level of protein expression by a lysine decarboxylase from *H. alvei* was higher than that of *E. coli* in all cellular fractions (Figure S1). This may explain the superior bioconversion ability of *H. alvei* LDC compared to *E. coli* LDC, although both enzymes are similar in size and amino acid sequence, showing 85% conserved residues between *E. coli* and *H. alvei* LDCs, 713, and 739 amino acids, respectively [50]. Based on these results, the lysine decarboxylase from *H. alvei* produced higher amounts of cadaverine due to its higher specific activity at 37 °C and higher level of expression in both soluble and insoluble fraction than the lysine decarboxylase from *E. coli*.

### 3.2. Development of IPTG-Free System for Whole Cell Bioconversion of l-Lysine into Cadaverine Using Lysine Decarboxylase from H. alvei and E. coli

The overall cost of production is the most important factor in the development of a whole cell bioconversion process for the commercial production of value-added chemicals. Process conditions, which may affect the economic feasibility of a whole cell bioconversion procedure are: (1) Conversion rate by biocatalyst; (2) the loading amount of substrate; and (3) the addition of inducers and enzyme co-factors to improve product formation [38,39,40,43]. In processes developed for the whole cell bioconversion of l-lysine into cadaverine, the addition of isopropyl β-d-1-thiogalactopyranoside (IPTG) and pyridoxal phosphate (PLP) is required for the efficient expression and the reaction of a lysine decarboxylase, because IPTG is used as an inducer for protein expression, and PLP is an important co-factor for LDC reactions [4,23,43]. However, the use of IPTG and PLP results in additional costs. Therefore, a whole cell biocatalyst system that does not require IPTG for the economical production of cadaverine was developed and evaluated for its ability for the whole cell bioconversion of l-lysine into cadaverine using a PLP-free medium. Firstly, we constructed an LDC expression system based on a pKE112 vector in order to develop an IPTG-free system for the expression of LDC from *H. alvei* and *E. coli.* The pKE112 vector system was previously used for the production of cadaverine and 5-aminovalerate from glucose [38,40]. Previously screened optimal host *E coli* XL1-Blue was used for the whole cell bioconversion experiments in this study [40]. The recombinant strains, XBHaLDC and XBEcLDC, were constructed for the IPTG-free expression of *H. alvei* and *E. coli* lysine decarboxylases, respectively. The protein expressions in the XBHaLDC and XBEcLDC strains in a PLP-free medium, with and without the supplementation of 0.05 mM of IPTG, were compared first. The levels of protein expression by both XBHaLDC and XBEcLDC were not significantly different after 24 and 96 h of incubation in a PLP-free medium with and without IPTG supplementation (Figure S2). Therefore, the bioconversion of l-lysine into cadaverine by these strains was compared in a PLP-free MR medium with 10 g/L of l-lysine [40]. The recombinant XBHaLDC strain produced 6.08 g/L of cadaverine from 10 g/L of l-lysine after 120 h (Figure 3). This value was 27% higher than the 4.78 g/L of cadaverine produced by XBEcLDC. Higher l-lysine into cadaverine conversion yield and productivity (0.05 g/Lh > 0.04 g/Lh) were obtained using the *H. alvei* l-lysine decarboxylase (87% molar yield) compared to the *E. coli* l-lysine decarboxylase (68% molar yield) (Figure 3) These results indicated that the pKE112 system may be used for the efficient conversion of l-lysine into cadaverine.

To further evaluate the potential of XBHaLDC for industrial application as a biocatalyst for whole cell bioconversion, the reaction was conducted in a PLP-free MR medium with a high substrate concentration (200 g/L (1.37 M) of l-lysine), which was supported by using industrial grade l-lysine. The whole cell bioconversion by XBEcLDC was compared to that of the XBHaLDC developed in this study. The recombinant XBHaLDC strain was capable of a higher rate of l-lysine consumption and cadaverine production than XBEcLDC in the early stages of reaction (Figure 4). The recombinant XBHaLDC strain exhibited a rapid conversion of l-lysine into cadaverine (123.2 g/L) at 12 h of reaction compared to the recombinant XBEcLDC strain (79.6 g/L). The measured productivity of XBHaLDC was significantly higher than XBEcLDC after 12 h of reaction (10.3 g/L/h > 6.6 g/L/h). The productivity of the XBHaLDC strain (10.3 g/Lh) was comparable to productivities (11.3–13.9 g/L/h) of other recombinant *E. coli* strains employed for the whole cell conversion of l-lysine to cadaverine in previous studies [41,42,43]. After 120 h of a whole cell bioconversion reaction using 200 g/L of l-lysine, XBHaLDC produced 136.3 g/L of cadaverine (97% molar yield), while XBEcLDC produced 132.8 g/L of cadaverine (95% molar yield). Though the final titer and molar yield of cadaverine by both strains were almost similar, it was noted that the rate of l-lysine conversion into cadaverine was faster with XBHaLDC than XBEcLDC. High productivity is useful because it reduces overall operating costs. Cadaverine produced by XBHaLDC was purified, concentrated, and polymerized with sebacic acid in order to investigate the material properties of bio-based polyamide 510 as previously described [35].

The polymerization of purified cadaverine with bio-based sebacic acid was conducted to investigate the use of a bio-based cadaverine monomer for the industrial scale synthesis of bio-based polyamide 510 [35]. As shown in Figure S4A, typical chemical shifts of polyamide 510 were detected. These results confirm that the polymer produced by the polymerization of bio-based cadaverine from the whole cell bioconversion of l-lysine and bio-based sebacic acid is polyamide 510. The material properties of polyamide 510 synthesized in this study were comparable to those reported in our previous study [35]. The relative viscosity was 2.7, the weight average molecular weight (*M_w_*) was 130,532 g/mol, and the number average molecular weight (*M_n_*) was approximately 78,412 g/mol. A DSC analysis of the resulting polyamide 510 revealed that the melting temperature (*T_m_*) and crystallization temperature (*T_c_*) were 215.7 and 171.1 °C, respectively (Figure S4B). The observed *T_m_* value of the synthetized polyamide 510 was comparable to that of polyamide 510 synthesized from chemical-grade cadaverine and bio-based sebacic acid. The value of the degradation temperature (*T_d_*) was 445.1 °C, indicating that the resulting polyamide 510 may be used as a heat resistant material (Figure S4C) [35]. Based on these results, the platform developed in this study for synthesizing polyamide 510 may be a potential replacement for petroleum-based polyamide.

## 4. Conclusions

In this study, we developed an IPTG- and PLP-free process for the whole cell bioconversion of l-lysine into cadaverine using a recombinant XBHaLDC biocatalyst to express a lysine decarboxylase from *H. alvei*. The enzyme characterization of the lysine decarboxylase encoded by *H. alvei* revealed that *H. alvei* LDC has higher protein expression levels and a higher specific activity at 37 °C than *E. coli* LDC, resulting in more the efficient production of cadaverine. The further purification of bio-based cadaverine produced via whole cell bioconversion and subsequent polymerization can produce polyamide 510 that may have a wider range of applications. Whole cell bioconversion using recombinant biocatalysts under optimized reaction conditions is a promising strategy which may be useful for the bio-based production of value added material, since the strategy developed in this study may be easily integrated into existing commercial l-lysine production processes. The overall cost of production is reduced due to separation of l-lysine production from the subsequent cadaverine conversion process, enabling easier separation and purification as well as a final fermentation derived cadaverine product of a higher purity. The further improvement of the strategy employed in our study may allow the co-production of cadaverine with other l-lysine-derived industrial chemicals of interest, such as 5-aminovalerate and glutaric acid [38,40].

## Figures and Tables

**Table 1:** Lists of bacterial strains and plasmids used in this study.

Strain/Plasmid	Relevant Characteristics	Ref.
**Strain**		
XL1-Blue	*recA1 endA1 gyrA96* thi-1 *hsdR17 supE44 relA1 lac* [FA1*proAB lacI^q^Z*ΔacI Tn10 (Tet^R^)]	[40]
BL21(DE3)	F- dcm ompT hsdS(rBmB-) gal λ(DE3)	[40]
XBHaLDC	*E. coli* XL1-Blue with pKE112-HaLdcC	This study
XBEcLDC	*E. coli* XL1-Blue with pKE112-EcLdcC	This study
BL21HaLDC	*E. coli* BL21(DE3) with pET22b-HaLdcC	This study
BL21EcLDC	*E. coli* BL21(DE3) with pET22b-EcLdcC	This study
**Plasmids**		
pKE112-MCS	Expression vector; *tac* promoter, *Ralstonia eutropha* PHA biosynthesis genes transcription terminator; Ap^r^	[40]
pKE112-EcLdcC	pKE112-MCS derivative, *tac* promoter, the *E. coli ldcC* gene, Ap^r^	This study
pKE112-HaLdcC	pKE112-MCS derivative, *tac* promoter, the *H. alvei ldc* gene, Ap^r^	This study
pET22b-EcLdcC	pET22b-MCS derivative, *T7* promoter, the *E. coli ldc* gene, Ap^r^	This study
pET22b-HaLdcC	pET22b-MCS derivative, *T7* promoter, the *H. alvei ldcC* gene, Ap^r^	This study
pET24ma-EcLdcC	pET24ma-MCS derivative, *T7* promoter, the *E. coli ldcC* gene, Km^r^	This study
pET24ma-HaLdcC	pET24ma-MCS derivative, *T7* promoter, the *H. alvei ldc* gene, Km^r^	This study

## Supplementary Materials

The following are available online at https://www.mdpi.com/2073-4360/11/7/1184/s1, Figure S1. SDS-PAGE analysis of recombinant *E. coli* BL21EcLDC and BL21HaLDC. Abbreviations: WCL—whole cell lysate, L—protein ladder, S—soluble fraction, IS—insoluble fraction; Figure S2. SDS-PAGE analysis of recombinant *E. coli* XBHaLDC and XBEcLDC after 96 h of cultivation in a PLP-free MR medium with 20 g/L of glucose and 10 g/L g l-lysine. Lanes: 1—E. coli XB wild type, MW- molecular weight marker, 2—XBEcLDC 24 h sample, 3—XBEcLDC 24 h sample with IPTG induction, 4—XBHaLDC 24 h sample, 5—XBHaLDC, 24 h sample with IPTG induction, 6—XBEcLDC 96 h sample, 7—XBEcLDC 96 h sample with IPTG induction, 8—XBHaLDC 96 h sample, 9—XBHaLDC 96 h sample with IPTG induction; Table S1. Primers used in this study. Figure S3. Decarboxylation of l-lysine into cadaverine by purified *E. coli* LDC and *H. alvei* LDC. Enzyme reactions were performed with the indicated concentration of l-lysine substrate (Symbols: filled blue circle, *E. coli* LDC; filled red circle, *H. alvei* LDC). Figure S4. Results of nuclear magnetic resonance spectroscopy (1H NMR spectra) (a) with detected solvent peaks [4.56–4.21 (multiplet, HFIP) and 7.27 (singlet, CDCl_3_)] and detected cadaverine backbone peaks [6.09 (2H, t; peak a), 3.27 (4H, q; peak b), 2.23 (4H, t; peak e), 1.58–1.54 (8H, m; peak c,f), 1.33 (10H, B; peak d,g)]; differential scanning calorimetry (DSC) (b); and thermogravimetric (TGA) analysis (c) of bio-based polyamide PA510. Table S1. Primers used in this study.
