# Supplementary material for: High-Level Conversion of l-lysine into Cadaverine by Escherichia coli Whole Cell Biocatalyst Expressing Hafnia alvei l-lysine Decarboxylase
Source: Polymers (Basel). 2019 Jul 14;11(7):1184. doi: 10.3390/polym11071184 (PMC6680443; doi:10.3390/polym11071184)
Supplement: Supplementary file 1 [file polymers-11-01184-s001.pdf]

polymers

MDPI

Supplementary File

High-Level Conversion of L-lysine into Cadaverine by *Escherichia coli* Whole Cell Biocatalyst Expressing *Hafnia alvei* L-lysine Decarboxylase

Hee Taek Kim[1,†], Kei-Anne Baritugo[2,†], Young Hoon Oh[1], Kyoung-Hee Kang[1], Ye Jean Jung[1,3], Seyoung Jang[1], Bong Keun Song[1], Il-Kwon Kim[4], Myung Ock Lee[5,6], Yong Taek Hwang[5], Kyungmoon Park[3], Si Jae Park[2*], Jeong Chan Joo[1]

1 Bio-Based Chemistry Research Center, Advanced Convergent Chemistry Division, Korea Research Institute of Chemical Technology, P.O. Box 107, 141 Gajeong-ro, Yuseong-gu, Daejeon 34114, Korea
2 Division of Chemical Engineering and Materials Science, Ewha Womans University, 52 Ewhayeodae-gil, Seodaemun-gu, Seoul 03760, Korea
3 Department of Biological and Chemical Engineering, Hongik University, 2639 Sejong-ro, Sinan-ri, Jochiwon-eup, Sejong-si 30016, Korea
4 Bioprocess R&D Center, DAESANG Corp., Icheon-si, Gyeonggi-do 17384, Korea
5 Lotte Chemical, 115 Gajeongbuk-ro, Yuseong-gu, Daejeon 34110, Korea
6 Department of Chemistry, KAIST, 291, Daehak-ro, Yuseong-gu, Daejeon 34141, Korea
* Correspondence: parksj93@ewha.ac.kr (S.J.P.), jcjoo@krict.re.kr (J.C.J.)
† Hee Taek Kim and Kei-Anne Baritugo contributed equally to this work.

polymers
MDPI

Figure S1. SDS-PAGE analysis of recombinant *E.coli* BL21EcLDC and BL21HaLDC. Abbreviations: WCL – whole cell lysate, L – protein ladder, S – soluble fraction, IS- insoluble fraction.

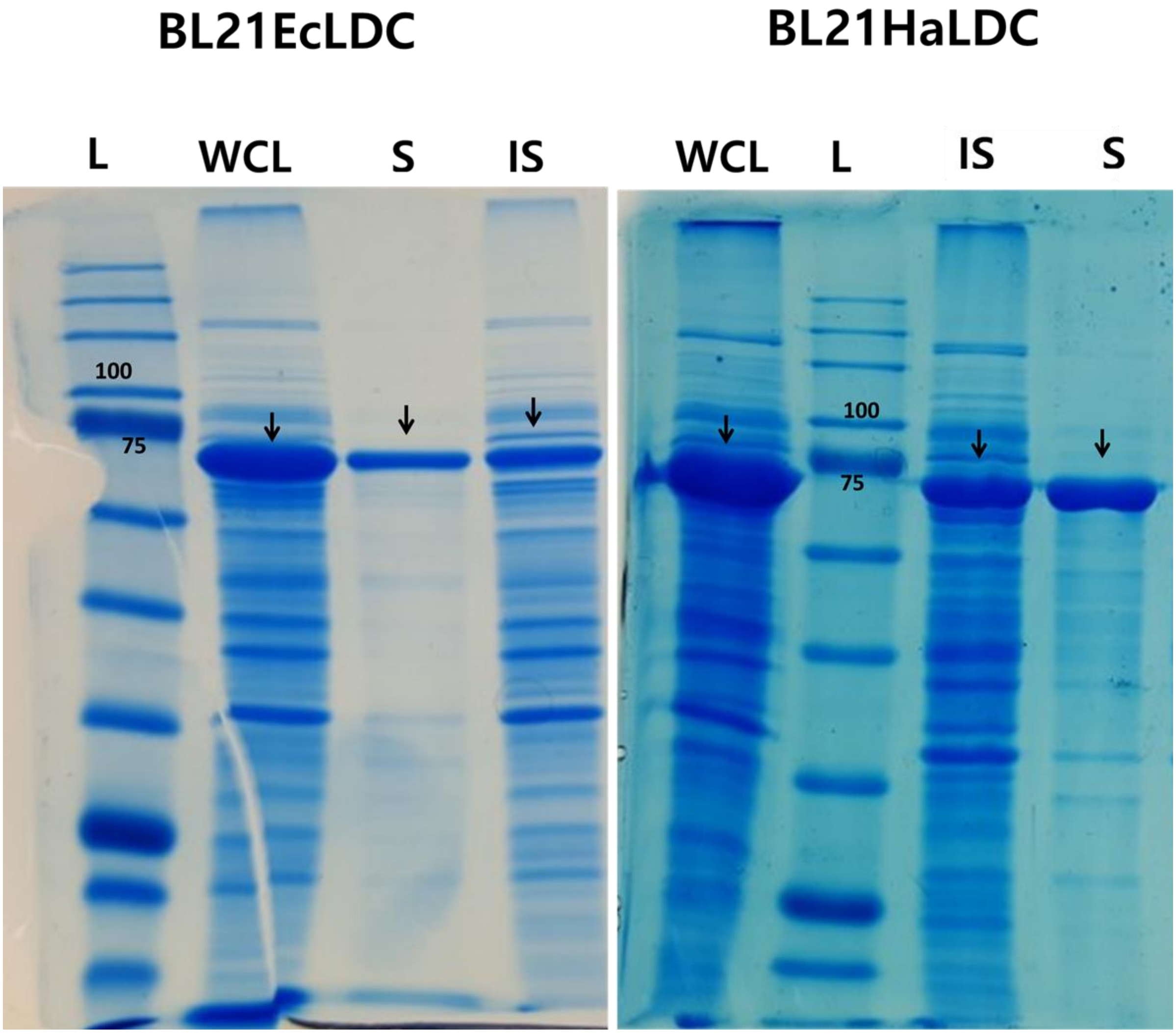

polymers

MDPI

Figure S2. SDS-PAGE analysis of recombinant *E.coli* XBHaLDC and XBEcLDC after 96 h of cultivation in PLP-free MR medium with 20 g/L of glucose and 10 g/L g L-lysine. Lanes: 1 – E. coli XB wild type , MW- Molecular weight marker, 2 – XBEcLDC 24 h sample, 3 – XBEcLDC 24 h sample with IPTG induction, 4 – XBHaLDC 24 h sample, 5 – XBHaLDC, 24 h sample with IPTG induction, 6 – XBEcLDC 96 h sample, 7 – XBEcLDC 96 h sample with IPTG induction, 8 - XBHaLDC 96 h sample, 9 – XBHaLDC 96 h sample with IPTG induction.

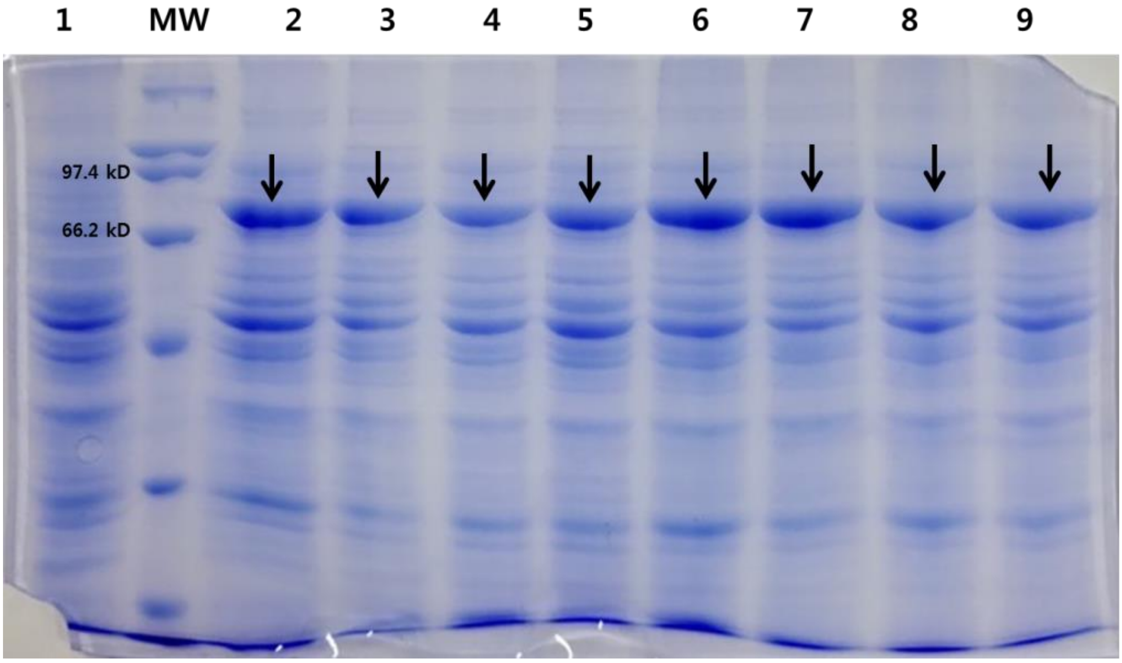

polymers

MDPI

Figure S3. Decarboxylation of L-lysine into cadaverine by purified *E. coli* LDC and *H. alvei* LDC. Enzyme reactions were performed with the indicated concentration of L-lysine substrate (Symbols: filled blue circle, *E. coli* LDC; filled red circle, *H. alvei* LDC).

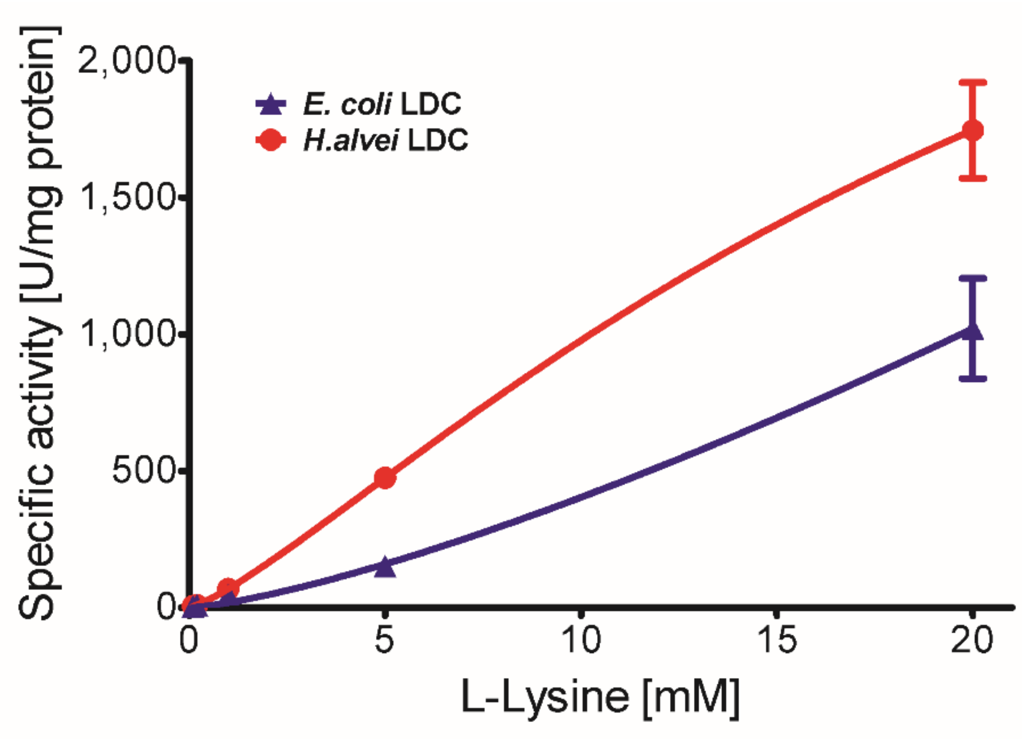

Figure S4. Results of nuclear magnetic resonance spectroscopy (1H NMR spectra) (a) with detected solvent peaks [4.56-4.21 (multiplet, HFIP) and 7.27 (singlet, $CDCl_3$)] and detected cadaverine backbone peaks [6.09 (2H, t; peak a), 3.27 (4H, q; peak b), 2.23 (4H, t; peak e), 1.58 -1.54 (8H, m; peak c,f), 1.33 (10H, B; peak d,g)] ; differential scanning calorimetry (DSC) (b); and thermogravimetric (TGA) analysis (c) of bio-based polyamide PA510.

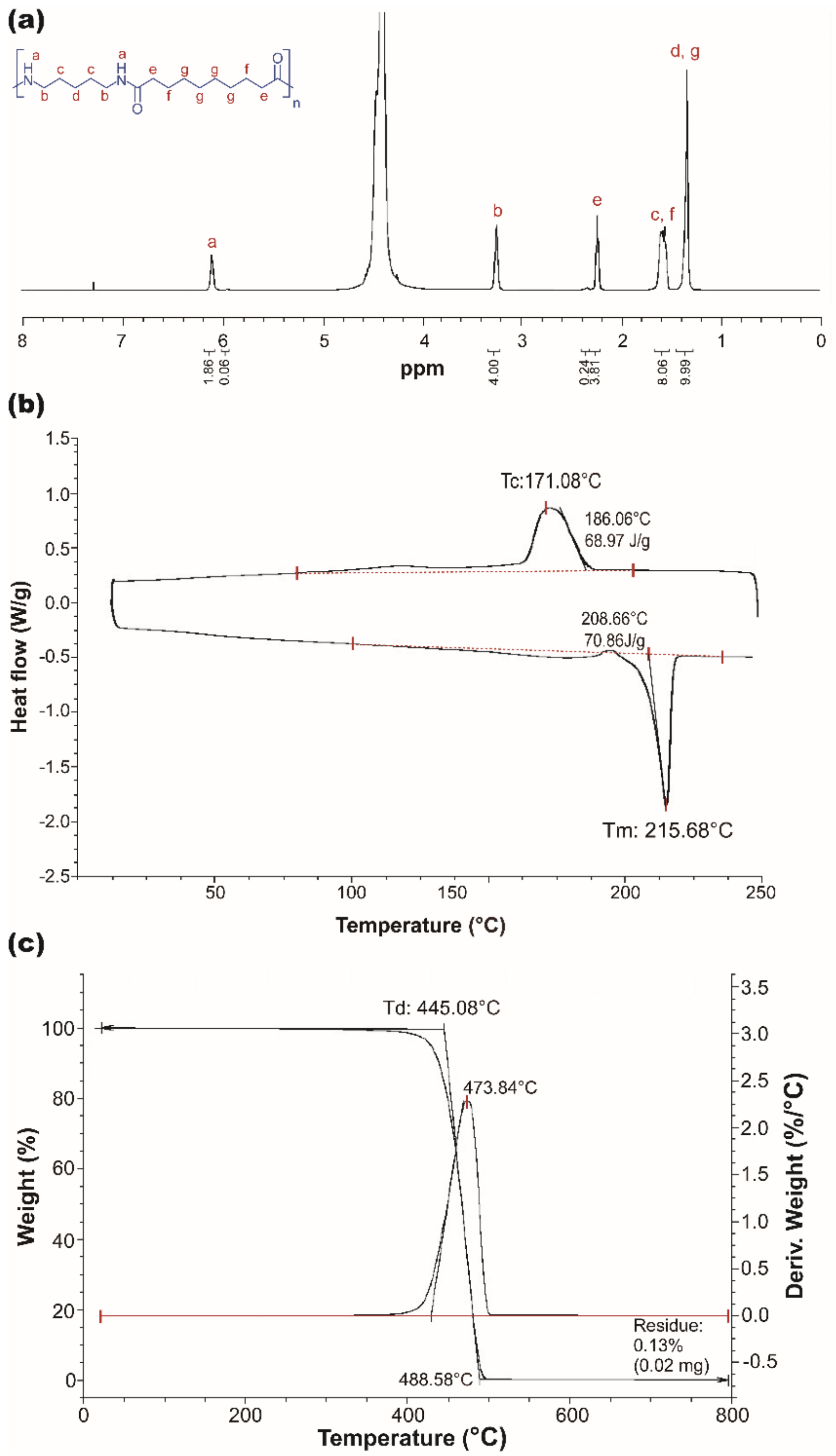

polymers

MDPI

Table S1. Primers used in this study.

Target Gene (Vector)	Primer	Primer sequence
ldcC from *E. coli* (pKE112-MCS)	F	GGATCC ATGAACATCATTGCCATTATGGGAC
	R	CCTGCAGG TCCCGCCATTTTTAGGACTCG
ldc from *H.alvei* (pKE112-MCS)	F	GGATCC ATGAATATCATTGCCATCATGAACG
	R	CCTGCAGG TGACTTCTTCGCCGCTGATG
ldcC from *E. coli* (pET22-MCS)	F	TCTAGA AATAATTTTGTTTAACTTTAAGAAGGAGATATACAT ATGAACATCATTGCCATTATGGGAC
	R	CTCGAG TCCCGCCATTTTTAGGACTCG
ldc from *H.alvei* (pET22-MCS)	F	TCTAGA AATAATTTTGTTTAACTTTAAGAAGGAGATATACAT ATGAATATCATTGCCATCATGAACG
	R	CTCGAG TGACTTCTTCGCCGCTGATG
ldcC from *E. coli* (pET24ma-MCS)	F	AAGCTT ATGAACATCATTGCCATTATGGGAC
	R	CTCGAG TCCCGCCATTTTTAGGACTCG
ldc from *H.alvei* (pET24ma-MCS)	F	AAGCTT ATGAATATCATTGCCATCATGAACG
	R	CTCGAG TGACTTCTTCGCCGCTGATG
